# Two New Alkaloids from *Narcissus serotinus* L.

**DOI:** 10.3390/molecules15107083

**Published:** 2010-10-13

**Authors:** Natalia B. Pigni, Strahil Berkov, Abdelaziz Elamrani, Mohammed Benaissa, Francesc Viladomat, Carles Codina, Jaume Bastida

**Affiliations:** 1 Department of Natural Products, Plant Biology and Soil Science, Faculty of Pharmacy, University of Barcelona, Barcelona, Spain; 2 Department of Chemistry, Faculty of Sciences Ain Chock, University Hassan II, Casablanca, Morocco

**Keywords:** *Narcissus serotinus*, Amaryllidaceae, alkaloids, narseronine, 1-*O*-(3´-acetoxy-butanoyl)lycorine

## Abstract

The Amaryllidaceae family is well known for the presence of an exclusive group of alkaloids with a wide range of biological activities. *Narcissus serotinus* L. is a plant belonging to this family and its geographical distribution is mainly located along the Mediterranean coast. In the present work, specimens collected near Casablanca (Morocco) were used to study the alkaloid content of this species. Starting with 350 g of the whole plant we used standard extraction and purification procedures to obtain fractions and compounds for GC-MS and NMR analysis. As well as five known alkaloids, we isolated two new compounds: 1-*O*-(3´-acetoxybutanoyl)lycorine and narseronine. The latter has been previously published, but with an erroneous structure.

## 1. Introduction

Plants belonging to the Amaryllidaceae family are well known for the presence of an exclusive group of alkaloids with a wide range of biological activities [1]. Within this group, the genus *Narcissus* has been extensively used in traditional medicine to treat a variety of health problems. Antiviral, antifungal and antitumoral activities are just some of the phamacological effects that have been proven for these alkaloids.

*Narcissus serotinus* L. is an autumn flowering species and the only member of the monotypic section *Serotini*. It grows mostly in calcareous sandy soil or maquis in dry coastal areas, and its geographical distribution extends over the coastal southern Mediterranean region, including southern Portugal, southern and eastern Spain, western and eastern Italy, Croatia, much of Greece and Israel, almost all the Mediterranean islands, north-west Morocco, Algeria, Tunisia and Libya [2,3].

The aim of this work is to investigate the alkaloid content of this species through the analysis of specimens collected in Morocco. In a previously published article, Vrondeli *et al.* [4] described the isolation of a new alkaloid from this species, suggesting a 3-epimacronine isomer. Based on the results reported herein, we propose an alternative structure, which also represents a new compound within the Amaryllidaceae alkaloid family.

## 2. Results and Discussion

**Figure 1:**
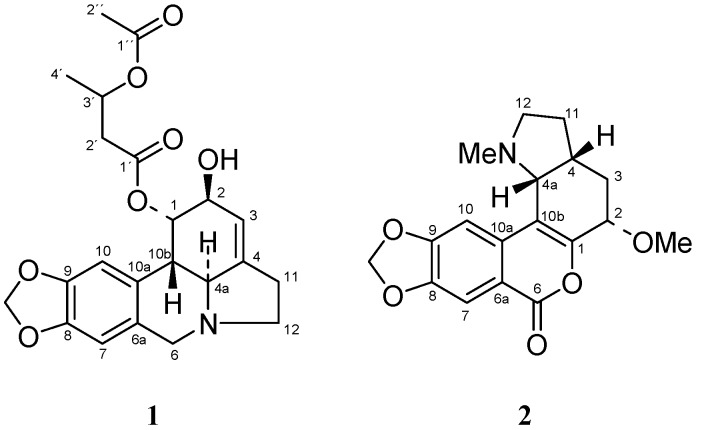
New alkaloids isolated from *N. serotinus* L. 1-*O*-(3´-acetoxybutanoyl)lycorine (**1**) and narseronine (**2**).

The MeOH extract of the fresh aerial parts and bulbs of *N. serotinus* L. was fractioned according to the methodology described in the experimental section. The GC-MS analysis of fraction B revealed the presence of lycorine. The analysis of fraction A showed a more complex mixture: in addition to lycorine [1,5] we determined the presence of galanthine [1,6], 1-*O*-(3´-hydroxybutanoyl)lycorine [7], assoanine [8] and hippeastrine [9] together with two new alkaloids (Figure 1). Identification of known compounds and structural elucidation of the new ones were achieved through the combined use of GC-MS, HRMS and one and two-dimensional NMR techniques.

The HRMS of **1** suggested a molecular formula C_22_H_26_NO_7_ for [M+H]^+ ^with a parent ion at *m/z* 416.1702 (calc. 416.1704). The EIMS showed a molecular ion [M]^+^ at *m/z* 415 (18%) with a base peak at *m/z* 226. It is interesting to note that the isomer 2-*O*-(3´-acetoxybutanoyl)lycorine, isolated from *Galanthus nivalis* [10], shows a very similar fragmentation pattern but with a base peak at *m/z* 250. However, the pattern observed for **1** shows the base peak at *m/z* 226 [7]. These empirical cases prove that the GC-MS technique is useful for differentiating between isomers with substituents at position 1 or 2. The ^1^H-NMR spectral data of compound **1** and the isomer, 2-*O*-(3´-acetoxybutanoyl)lycorine, are very similar too, showing the major difference in proton shielding at positions 1 and 2: in **1 **H-1 is more deshielded (δ 5.68) than the same proton in the isomer (δ 4.51) and the inverse situation occurs for H-2, which appears at δ 4.23 in the spectrum of **1** and at δ 5.31 for the isomer. Considering its coupling constant values, we assume that the configuration of **1** is the same as that proposed for 2-*O*-(3´-acetoxybutanoyl)lycorine and 1-*O*-(3´-hydroxybutanoyl)lycorine. The high coupling constant (10.4) observed between H-4a and H-10b suggests a *trans-*diaxial configuration. Protons 6β and 12β are more deshielded than 6α and 12α, respectively, because of the *cis-*lone pair of the nitrogen atom. The combined data suggested for compound **1 **the structure of 1-*O*-(3´-acetoxybutanoyl)lycorine. The ^1^H-NMR, COSY and HSQC data are recorded in Table 1. 

**Table 1:** ^1^H-NMR, COSY and HSQC data of 1-*O*-(3´-acetoxybutanoyl)lycorine (**1**).

Position	^1^H δ (J in Hz)	COSY	HSQC
1	5.68 *s*	H-2, H-10b	72.5 *d*
2	4.23 *dt* (3.3, 1.7)	H-1, H-3, H-11	69.4 *d*
3	5.56 *m*	H-2, H-11	116.9 *d*
4a	2.76 *d* (10.4)	H-10b	61.9 *d*
6α	3.54 *d* (14.1)	H-6β	56.6 *t*
6β	4.16 *d* (14.1)	H-6α	56.6 *t*
7	6.58 *s*		107.3 *d*
10	6.72 *s*		104.8 *d*
10b	2.91 *d* (10.4)	H-1, H-4a	38.8 *d*
11 (2H)	2.65 *m*	H-2, H-3, H-12α, H-12β	28.4 *t*
12α	2.42 *dd* (9.3, 5.0)	H-11, H-12β	53.4 *t*
12β	3.38 *dt* (9.2, 4.8)	H-11, H-12α	53.4 *t*
OCH_2_O	5.92 *s*		100.8 *t*
2´_A_	2.43 *dd* (15.5, 5.4)	H-2´_B_, H-3´	40.5 *t*
2´_B_	2.53 *dd* (15.5, 7.8)	H-2´_A_, H-3´	40.5 *t*
3´	5.10 *m*	H-2´_A_, H-2´_B_, H-4´	66.9 *d*
4´	1.14 *d* (6.3)	H-3´	19.3 *q*
AcO (2´´)	1.95 *s*		20.7 *q*

The HRMS analysis of narseronine (**2**) suggested a molecular formula C_18_H_20_NO_5 _for the parent ion [M+H]^+^ at *m/z* 330.1340 (calc. 330.1336). This indicates a molecular formula C_18_H_19_NO_5_, in accordance with a molecular weight of 329. The EIMS showed a molecular ion [M]^+^ at *m/z* 329 (20%). The mass spectral fragmentation pattern is not similar to those commonly shown by the homolycorine type compounds, because of the absence of a double bond between C-3 and C-4. The unusual occurence of a double bond at position C-1/C-10b probably drastically changes this pattern. Its ^1^H NMR spectrum exhibited two singlets at δ 7.66 and 7.29 for the *para-*oriented aromatic prontons H-7 and H-10, respectively, with H-7 more deshielded due to the *peri-*carbonyl group [1]. Also, the NOESY experiment showed the spatial proximity between H-10 and the *N*-methyl group. Two doublets appeared at δ 6.10 and δ 6.12 for the protons of the methylendioxy group. A triplet at δ 4.22 was assigned to H-2, coupled to H-3 with a *J* = 6.1 Hz, suggesting an equatorial orientation with a similar dihedral angle between H-2 and the two H-3 protons; this is consistent with the α position of the methoxy group at C-2 and with the NOESY correlation of this substituent with H-11. A doublet at δ 3.94, was undoubtedly assigned to H-4a for a 3JC-H HMBC correlation with the *N*-methyl group; COSY experiment showed its only correlation with H-4, with a *J* = 6.4 Hz suggesting a *cis-* C/D ring fusion [11,12]. The spectrum also showed, between the most significant signals, a singlet integrating for 3 protons at δ 3.57 assigned to the methoxy group at C-2, a doublet of triplets at δ 3.05, assigned to H-12α, more deshielded than H-12β because of the *cis-*lone pair of the nitrogen atom [1], a singlet corresponding to the *N*-methyl group at δ 2.41, also supporting the *cis-* C/D ring junction if we consider the empirical correlations of *N*-methyl chemical shifts with stereochemical assignments suggested by Jeffs *et al.* [11]; and a doublet of triplets at δ 2.01 assigned to H-3α, showing spatial proximity with the *O*-CH_3_ group in the NOESY experiment. The NMR spectral data is shown in Table 2.

Narseronine was previously isolated by Vrondeli *et al.* [4] but published with an erroneous structure. They suggested a 3-epimacronine isomer, a tazettine type alkaloid but their mass spectral fragmentation proposal does not explain the occurrence of the most abundant peaks of the mass spectrum, such as *m/z* 240 or 241. Also, the ^1^H-NMR assignment is not adequate, including, for instance, the protons in an α-position to the *N*-methyl group (H-6 in their numbering system) at δ 2.30-1.80 ppm, a more shielded displacement than can be expected for a proton in such an electronic environment.

**Table 2:** ^1^H-NMR, COSY, NOESY, ^13^C-NMR (HSQC) and HMBC data of narseronine (**2**).

Position	^1^H δ (J in Hz)	COSY	NOESY	^13^C δ	HMBC
1	-	-	-	152.9 *s*	-
2	4.22 *t* (6.1)	H-3α, H-3β	H-3α, H-3β, OCH_3_	74.9 *d*	C-1, C-3, C-4, C-10b, OCH_3_
3α	2.01 *dt* (13.5, 5.5)	H-2, H-3β, H-4	H-2, H-3β, H-4, OCH_3_	31.4 *t*	C-1, C-2, C-4, C-4a, C-11
3β	2.22 - 2.13 *m(overlapped)*	H-2, H-3α, H-4	H-2, H-3α, H-4, OCH_3_	31.4 *t*	C-1, C-2, C-4, C-4a, C-11
4	2.64 *m*	H-3α, H-3β, H-4a, H-11α, H-11β	H-3α, H-3β, H-4a, H-11α, H-11β	35.1 *d*	C-12
4a	3.94 *d* (6.4)	H-4	H-4, H-10, NCH_3_	61.6 *d*	C-1, C-3, C-4, C-10a, C-10b, C-11, C-12, NCH_3_
6	-	-	-	161.5 *s*	-
6a	-	-	-	116.4 *s*	-
7	7.66 *s*	-	-	107.8 *d*	C-6, C-8, C-9, C-10a
8	-	-	-	148.4 *s*	-
9	-	-	-	153.8 *s*	-
10	7.29 *s*	-	H-4a, NCH_3_	103.3 *d*	C-6a, C-8, C-9, C-10b
10a	-	-	-	135.1 *s*	-
10b	-	-	-	110.8 *s*	-
11α	2.22 - 2.13 *m(overlapped)*	H-4, H-11β,	H-4, H-11β, H-12α, H-12β, OCH_3_	29.6 *t*	C-3, C-4a
		H-12α, H-12β			
11β	1.90 *ddd* (12.6, 8.3, 4.2)	H-4, H-11α, H-12α, H-12β	H-4, H-11α, H-12α, H-12β, OCH_3_	29.6 *t*	C-3, C-4a
12α	3.05 *dt* (11.0, 7.6)	H-11α, H-11β, H-12β	H-11α, H-11β, H-12β, NCH_3_	54.3 *t*	C-4, C-4a, C-11, NCH_3_
12β	2.81 *m*	H-11α, H-11β, H-12α	H-11α, H-11β, H-12α, NCH_3_	54.3 *t*	C-4, C-4a, C-11, NCH_3_
OCH_2_O	6.10 *d* (1.2) 6.12 *d* (1.2)	-	-	102.4 *t*	C-8, C-9
OCH_3_	3.57 *s*	-	H-2, H-3α, H-3β, H-11α, H-11β	58.3 *q*	C-2
NCH_3_	2.41 *s*	-	H-2, H-3α, H-3β, H-11α, H-11β	41.8 *q*	C-4a, C-12

## 3. Experimental

### 3.1. General

NMR spectra were recorded in a Mercury 400 MHz or a Varian VXR 500 MHz, instrument using CDCl_3_ as the solvent and TMS as the internal standard. Chemical shifts were reported in δ units (ppm) and coupling constants (*J*) in Hz. EIMS were obtained on a GC-MS Agilent 6890 + MSD 5975 operating in EI mode at 70 eV. A HP-5 MS column (30 m × 0.25 mm × 0.25 μm) was used. The temperature program was: 100-180 ºC at 15 ºC min^-1^, 1 min hold at 180 ºC, 180-300 ºC at 5 ºC min^-1^ and 1 min hold at 300 ºC. Injector temperature was 280 ºC. The flow rate of carrier gas (Helium) was 0.8 mL min^-1^. In most cases the split ratio was 1:20, but with more diluted samples a split ratio of 1:5 was applied. UV spectra were obtained on a DINKO UV2310 instrument and IR spectra were recorded on a Nicolet Avatar 320 FT-IR spectrophotometer.

### 3.2. Plant material

Whole plants of *Narcissus serotinus* L. (Amaryllidaceae) were collected in October 2009 during the flowering period in Ben Slimane, near Casablanca (Morocco), and identified by Professor El Ghazi. A voucher sample (MB-026/2009) was deposited at the Herbarium of the Faculty of Sciences Ain Chock, University Hassan II.

### 3.3. Extraction and isolation of alkaloids

The fresh whole plant (350 g) was crushed and extracted with methanol (1 × 800 mL, 24 h; 1 × 800 mL, 72 h; and 2 × 400 mL, 48 h each). The extract was evaporated under reduced pressure to yield 5.5 g. This crude extract was dissolved in 100 mL of H_2_SO_4_ 1% (v/v) and neutral material was removed with Et_2_O (6 × 100 mL). The acidic soln. was basified with 25% ammonia up to pH 9-10 and extracted with EtOAc (3 × 100 mL) to give extract A (149.4 mg). Another extraction with EtOAc (2 × 100 ml) gave extract B (23 mg). Both fractions were dried with anhydrous Na_2_SO_4_, filtered and completely dried under reduced pressure. Referred to the fresh weight, the sum of these two extracts represents approximately 0.05%. After dissolving A and B in MeOH, lycorine crystallized directly. Extract A was subjected to a vacuum liquid chromatography (VLC) [13] using a silica gel 60 A (6-35 μ) column with a diameter of 1 cm and a height of 4 cm. Alkaloids were eluted using hexane gradually enriched with EtOAc, and then EtOAc gradually enriched with MeOH. Fractions of 10 mL were collected (105 in total) monitored by TLC (Dragendorff´s reagent, UV 254 nm) and combined according to their profiles. Five main fractions were obtained and subjected to preparative TLC (20 cm × 20 cm × 0.25 mm, silica gel 60F254). Narseronine (**2**, 4.5 mg) and 1-*O*-(3´-acetoxybutanoyl)lycorine (**1**, 1.5 mg) were obtained in major quantities from fractions *32-38* (eluted from VLC with hexane-EtOAc, 30:70 to 20:80) through preparative TLC (EtOAc-hexane 4:1 + 25% ammonia).

*1-O-(3´-Acetoxybutanoyl)lycorine* (**1**). UV (MeOH) λ_max_ nm: 368.0, 260.0. IR (CHCl_3_) ν_max_ cm^-1^: 2959, 2924, 2853, 1735, 1461, 1371, 1244, 1170, 1038, 776. ^1^H-NMR, COSY, HSQC (400 MHz, 500 MHz, CDCl_3_) see Table 1. EIMS 70eV (rel. int.): 416 (4), 415 (18), 354 (1), 269 (12), 268 (37), 250 (27), 227 (75), 226 (100), 192 (4), 147 (5), 96 (4), 69 (15), 43 (27). HRMS of [M+H]^+^ *m/z* 416.1702 (Calc. 416.1704 for C_22_H_26_NO_7_). 

*Narseronine* (**2**). UV (MeOH) λ_max_ nm: 322.5, 286.0, 241.5. IR (CHCl_3_) ν_max_ cm^-1^: 2928, 1718, 1503, 1482, 1415, 1283, 1256, 1162, 1106, 1035, 935, 754. ^1^H-NMR, COSY, NOESY, HSQC, HMBC and ^13^C-NMR (500 MHz, CDCl_3_) see Table 2. EI-MS 70eV (rel. int.): 329 (20), 328 (21), 314 (2), 299 (28), 272 (38), 271 (18), 256 (46), 255 (16), 254 (30), 242 (34), 241 (98), 240 (100), 228 (13), 213 (18), 212 (13), 59 (42), 57 (60), 44 (37). HRMS of [M+H]^+^ *m/z* 330.1340 (Calc. 330.1336 for C_18_H_20_NO_5_). 

## 4. Conclusions

These results lead us to conclude that *N. serotinus* L. is an interesting source of alkaloids with potential pharmacological activities. Lycorine type alkaloids have shown notable properties as potent antimalarial and antitrypanosomal agents [7]. Recent investigations, including structure-activity studies, have also demonstrated they are potent inducers of apoptosis with good antitumoral activities [5,14]. In this sense, 1-*O*-(3´-acetoxybutanoyl)lycorine (**1**) is an attractive candidate for research in these areas. The isolation of narseronine (**2**) is also promising; this is the first report of a double bond between C-1 and C-10b in a homolycorine type structure, a feature that confers rigidity to the portion of the molecule formed by A-B rings, and also has a stabilising effect due to the extended conjugated system. This could be an interesting characteristic for potential biological activities related with pharmocophores with such requirements. In other respects, previous reports of antifungal activity of homolycorine-related structures such as hippeastrine [15], suggest narseronine has potential activity as an antifungal agent.
